# Clinical realism: a new literary genre and a potential tool for encouraging empathy in medical students

**DOI:** 10.1186/s12909-015-0372-8

**Published:** 2015-07-03

**Authors:** Paula McDonald, Katy Ashton, Rachel Barratt, Simon Doyle, Dorrie Imeson, Amos Meir, Gregoire Risser

**Affiliations:** 1 Clinical Lecturer Manchester Medical School, Manchester, England; 2 Medical student Manchester Medical School, Manchester, England

**Keywords:** Medical education, Medical humanities, Creative writing, Empathy, Affinity, Clinical realism

## Abstract

**Background:**

Empathy has been re-discovered as a desirable quality in doctors. A number of approaches using the medical humanities have been advocated to teach empathy to medical students. This paper describes a new approach using the medium of creative writing and a new narrative genre: clinical realism.

**Methods:**

Third year students were offered a four week long Student Selected Component (SSC) in Narrative Medicine and Creative Writing. The creative writing element included researching and creating a character with a life-changing physical disorder without making the disorder the focus of the writing. The age, gender, social circumstances and physical disorder of a character were randomly allocated to each student. The students wrote repeated assignments in the first person, writing as their character and including details of living with the disorder in all of their narratives. This article is based on the work produced by the 2013 cohort of students taking the course, and on their reflections on the process of creating their characters. Their output was analysed thematically using a constructivist approach to meaning making.

**Results:**

This preliminary analysis suggests that the students created convincing and detailed narratives which included rich information about living with a chronic disorder. Although the writing assignments were generic, they introduced a number of themes relating to illness, including stigma, personal identity and narrative wreckage. Some students reported that they found it difficult to relate to “their” character initially, but their empathy for the character increased as the SSC progressed.

**Conclusion:**

Clinical realism combined with repeated writing exercises about the same character is a potential tool for helping to develop empathy in medical students and merits further investigation.

## Background

We live in a post-modern society where a technocratic approach to medicine is no longer considered sufficient or desirable. Twenty-first century patients are looking for person-centred care: they want to be listened to and to have a dialogue with their doctor, to be healed rather than cured [1, 2]. The once-forgotten quality of empathy has been re-discovered as a desirable quality in doctors, and although it has been suggested that there are situations where objectivity may be more important [3, 4], empathy has been shown to be associated with improved patient satisfaction and better clinical outcomes [5–8].

### What is empathy?

Although most people would claim to be able to recognise empathy, there is no agreed definition. One definition is “psychological processes that make an individual have feelings that are more congruent with another’s situation than with his or her own” [9]. Most definitions include the ability to understand and share others’ feelings, and are sometimes divided into two components, affective and cognitive. **Cognitive empathy**, also referred to as perspective-taking, is the ability to understand how another person feels and what they might be thinking. The second component of empathy, **affective empathy**, includes experiencing the emotions that the other person is feeling [9]. Some observers add an intention to help [10]. A related concept is affinity, our natural attraction to others. Larson and Yao have commented on the tendency we have to empathise with people who are like us, and to not empathise with people who are seen as “other” or different from us [11, 12].

Neurobiological correlates of empathy have been demonstrated. A meta-analysis of neuro-imaging research on empathy found that the dorsal part of the left anterior midcingulate cortex was found to be activated more frequently in the cognitive- evaluative form of empathy, while the right anterior insula was activated only in the affective-perceptive form of empathy [13]. Recent, though disputed research, suggests that “mirror neurons” may be “the neural basis of our empathic capacities” [14, 15].

A number of approaches using the medical humanities have been advocated for teaching empathy to medical studentsx [16]. This paper describes a new approach using the medium of creative writing and a new narrative genre: “clinical realism”.

### What is clinical realism?

Clinical realism has its roots in the realism movement in art and literature in the nineteenth century. Realism, notes Morris, is “a literary form that has been associated with an insistence that art cannot turn away from the more sordid and harsh aspects of human existence” [17]. Modernism aimed to render a full report of human experience through the use of details, and was portrayed by George Eliot as “old women scraping carrots with their work-worn hands” but also extended the subject matter of novels to incorporate class, gender, sexuality, and social reality [17, 18]. Although it was not seen as a separate movement at the time, a number of novelists such as Balzac, Flaubert, Eliot and Zola included realistic medical content in their novels. Rothfield analysed a selection of British and French medical realist novels, and noted that although realism is often equated with representation, the connection between medicine and realism varied with the purpose and period of the author. For example, he notes that the smallpox contracted by Mme. de Merteuil in *Les Liasions Dangerouses* serves as a metaphor which offers moral, social and narrative closure: “her disease had turned her round and… now her soul is in her face.” We are told that she has been disfigured and has lost an eye, but there is no more detail about the disease. In contrast, Zola goes into graphic detail to discuss the smallpox which has killed his heroine in *Nana,* and which also perhaps, serves as a metaphor for the degeneration of an entire society: “the pustules had invaded the whole face, so that one pock touched the next. Withered and shrunken, they had taken on the greyish colour of mud…” Rothfield argues that medicine provided novelists with narrative strategies, epistemological assumptions and models of professional authority, and early medical realist novels in particular tended to portray illness as “either a fundamental ontological predicament or a punctual sign of innate moral inadequacy” [18].

Realism was replaced by other movements, including anti-realism, naturalism, detective fiction, modernism and post-modernism. In 1926, Virginia Woolf wrote an essay “On being ill”, commenting: “Considering how common illness is...... it becomes strange indeed that illness has not taken its place with love, battle and jealousy among the prime themes of literature” [19]. As Jurecic notes, the paucity of writing about illness seems even more remarkable when it is set against the fact that five per cent of the world's population had died in the flu epidemic of 1918/19, less than a decade before Woolf wrote her essay [20].

Frank has commented on the rise of modernism, when “the medical chart became the official story of the illness”. He suggests that in post-modern time, people reclaimed the capacity for telling their own story [21]. Jurecic notes the flood of illness narratives that appeared in response to the emergence of HIV/AIDs in the late twentieth century, and the establishment of illness/disability narratives as a literary genre at around this time, albeit one that is not taken very seriously by critics [20]. The last decade has seen a similar explosion of graphic medicine accounts of illness. Graphic medicine explores the interaction between the medium of comics and the discourse of healthcare, and is allied to the graphic novel movement, which has produced “serious” comic books, aimed at adults. There are now numerous graphic medicine novels and autobiographies which recount illness narratives in cartoon form [22, 23]. There remains, however, very little representation of physical health problems in literary fiction. ONS statistics for 2014 show that over 11 million people in the UK have a limiting long-term illness, impairment or disability [24] QoF (Quality and Outcomes Framework) statistics from GP practices for 2012/13 show that 13.7 % of registered patients have hypertension, 6.2 % have diabetes, 5.9 % have asthma, 4 % have chronic kidney disease, 3.3 % have coronary heart disease, 1.8 % have chronic obstructive pulmonary disease, 1.7 % have had a stroke or TIA and 1.6 % have atrial fibrillation [25]. And yet people with chronic physical health problems are almost invisible in contemporary fiction - we rarely see anyone adjusting their dose of insulin, having side-effects from their medication, or plotting the route of an outing by the proximity of available toilets.

Illness and disability can profoundly affect an individual's life opportunities and personal identity [24]. Goffman has written about how people with stigmatising conditions, including many health conditions, have to manage their “spoiled identity” [26]. Sontag notes how diseases such as cancer, TB and syphilis are seen “as punishment”, betraying a character flaw, while the names of these diseases themselves have become adjectival, being used as a metaphor for evil [27]. Kleinman comments: “The trajectory of chronic illness assimilates to a life course, contributing so intimately to the development of a particular life that illness becomes inseparable from life history” [28]. And yet in contemporary fiction we may hear about a character's appearance, class, ethnicity, education, job, politics, sexual proclivities, taste in music and even the contents of their handbag, but the day -to -day experience of living with a health problem is rarely represented.

For the purposes of this course, one of the authors (PM) created a new genre, clinical realism. This is defined as “*Fictional writing where health problems are systematically represented, not as a metaphor, not as a plot point, and not as the central topic of the writing, but as a part of a character's personal identity and day to -day experience*”.

## Methods

The creative writing course ran at Manchester Medical School from 2010–2013 and was offered to third year students twice a year as part of a four-week SSC in Narrative Medicine and Creative Writing. It accepted 6 to 8 students, who created and wrote about a character with a life-changing physical disorder. The students created a character with a back story including a serious health problem or caring for someone with a serious health problem. They then “inhabited” their character for four weeks, writing all their assignments in the first person. The assignments were not about their health problem, but were written in a “clinical realism” style, which, where possible, introduced some details about how they lived with their disorder into each piece of writing. They also wove details of the history of their disorder into their stories where it was relevant. The writing was mainly done as “homework”, and was discussed in a weekly workshop, where students were asked to comment separately on the creative writing and the medical realism aspects of each submission. At the end of the course, the students wrote obituaries for their characters.

The course evolved from 2010–2013 in response to student feedback and teacher/learner experience. In 2013, the cohort on which this paper is based had workshops on narrative medicine [29] and transactional analysis [30], and interviewed patients with chronic disorders and listened to their stories. They presented one group workshop on a narrative medicine topic during the course, and at the end of the SSC, they wrote an essay on an aspect of narrative medicine, which could incorporate examples of their own writing if they wished. The last cohort of creative writing students also wrote about how they had developed their characters and their reflections on the course.

The creative writing sessions were developed by PM from part of a teaching module from an MA in creative writing at Manchester Metropolitan University. To create their characters, the students initially chose two letters, which became the initials of their character’s name. They then drew randomly from a selection of paper slips with diagnoses on - in this cohort, irritable bowel syndrome, post head injury with memory impairment, post-treatment breast cancer, rheumatoid arthritis, hepatitis C and type 1 diabetes. They drew further slips to determine their age, sex, what sort of accommodation they lived in and who they lived with. They then chose names for their characters and completed a character creation questionnaire, which included details of their character, including details of their disorder and how it affected them, and also other aspects such as their appearance, what sort of clothes they wore, where they did their shopping, hobbies, aspirations, regrets and how they saw their future. The students were encouraged to research their characters by looking at online patient groups and blogs.

The workshop and writing exercises included dialogue, plot, genre, arc of the plot, description, unreliable narrator and microfiction exercises. As an example of the content, the genre workshop consisted of a discussion about genres, a short film parodying different genres, an exercise where the students wrote “consequences” type stories, each taking it in turn to write five minute segments of a “genre” story about each of their characters, and a homework genre exercise based on a conflict to do with eating lunch in a busy café.

There were some interactions with the narrative medicine seminars, for example the discussions of plot and genre in creative writing were followed by discussions and excises on plot and genre in narrative medicine.

As this report does not include real patient details and the module was approved by Manchester Medical School, no ethical approval was neccessary.

### Theoretical approaches

This article focuses on the creative writing outputs of the cohort of six students attending the last session of the course. Additional material written by the students about their experiences of developing their characters and relevant comments from the end of module essays were also analysed with their written permission. The students were aged 21–24, three were female and three male, and they defined themselves as four white British, one white Other and one Other. Two were post graduate students and one was an Erasmus student [31] from France, spending a year at a British medical school.

The student outputs were analysed thematically using a constructivist approach to meaning-making as a guiding principle, as developed by Charmaz [32] and as used by Kristiansson et al. [33] Constructivism asserts that reality is constructed by individuals as they assign meaning to the world around them. It aims to incorporate the multiple voices, views and visions of participants in rendering their lived experiences, and seeks to identify the patterns of behaviour in which people engage, and to understand them rather than explain them [32].

The outputs were analysed to see how successfully the students had been able to “stand in someone else's shoes.” In particular, evidence of representation of day- to- day experience of living with a chronic disorder and of its effects on personal and social identity were sought, and the students’ comments on the course were analysed.

## Results

### Approaches to creating character

The students’ approaches to creating their characters varied. One student based his character on patients he has seen previously whose stories had resonated with him, explaining;

*My first instinct was to rush home to my textbooks to look up staging and prognoses, experimental treatments and side effects – to research the pathology to its fullest extent. However, I soon realised that this approach would give a medicalised perspective of the situation. It would be the narrative of a health professional rather than that of a patient. Instead, I consulted my patient logs…” (student 5)*

Another reported that the exercise had made him look back and reflect on the patients he had met previously.

*I guess I was also influenced by patients from my previous clinical placements at the hospital and in the community. From their stories and how they live with their condition. This writing was a great look back and reflection on them, on how diseases and diagnoses can affect you and your life. (student 6)*

Others researched patient blogs and self- help sites as well as academic articles.

*I read health discussions forums to read different patient’s stories, to have a better idea, from their point of view of what their concerns are, what helped them. I then applied it to my character to see what he could have been experiencing. (student 3)*

*My character development was aided by reading online patient blog entries and discussion groups written by people suffering with Hepatitis C. This online resource allowed me to gain an insight into the daily struggles faced by real people living with hepatitis C, from drug side-effects to stigma faced - helping me to create a more informed and believable narrative. These online health narratives also helped me to gain an insight into the tone and language used by patients to describe their experiences, which I tried to incorporate into my own writing. (student 4)*

One student set herself the task of imagining living with IBS in a different culture.

*“It is estimated that almost 30 % of the Nigerian population have IBS, compared to 10-20 % of the western population. In my creative writing, I wanted to convey the cultural and social misconceptions of IBS in different cultures and the difficulties people with the condition face.*

*To do so, I made Kazoo an isolated character who lived in a small fictional village in the north of Nigeria who is aware of something not being "quite right" with his health but misunderstands the source of the problems”. (student1)*

A student researching head injury pointed out that almost all the accounts she came across were written from the point of view of friends and family:

*My research spanned numerous academic journals and texts, each of which provided interesting pathological insights, and a number of charitable websites aimed at sufferers of such injury – or rather their friends and family. The voice of the patient themselves was notably absent but rather their story was told in varying styles by those involved in their care. (student 2)*

### Writing about the day- to- day experiences of living with a chronic disorder

Students were instructed to incorporate small details of living with chronic disorders, and did so successfully.

*2 pm. Time to check my blood sugar (student 3)*

*I sigh and finish my coffee. I’m thinking of the stairs to the apartment (student 5)*

*I lay my burdens upon it (the kitchen table), the keys atop the jumbled letters, my handbag beside the bag of groceries. What now? What should be done next?*

*I regard my collection and try to remember the next step in the sequence. The letters have confused my routine, my daughter normally takes care of them. (student 2)*

Facebook update:

*Really not coping well today. Everything against me- boiler broke down last night, missed the postman, and doctor cancelled the clinic. ARGHHHHH. Any suggested pick me ups/hugs/film recommendations- send this way!*

*Like Comment Share Posted 27 min ago (student 4)*

*I finally find a pair of empty seats, but my victory is quickly dashed at the next stop as a huge, pungent man gets on, occupying the seat next to me. I look out of the window as waves of nausea hit me. Can't tell if it's the giant's odour or a parting gift from the chemo. Maybe it's both. (student 5)*

### Illness-related themes covered in student writing

Although the writing exercises were generic, students nevertheless introduced a number of important illness-related themes, in particular in relation to stigma, personal identity and narrative wreckage.

### Narratives dealing with stigma

Goffman suggested that stigmatised people have culturally unacceptable traits that are not considered “normal”. Stigmatised people are considered to have a “spoilt” identity. Different medical conditions are viewed as more or less stigmatising. He suggests that the stigmatised person suffers from status loss and discrimination, and is expected to follow certain rules for handling “normals” (non- stigmatised people). These include not responding to snubs and insults, allowing intrusive questions and agreeing to be helped [26].

Here a character with diabetes makes a challenging remark, which is dismissed by her brother:

*Have you had the right dose of insulin? You know you can get… difficult if you haven’t. (student 3)*

A character who has been living on the streets is rejected:

*“Get that piece of filth out of my sight!” (student1)*

A neighbour feels able to ask intrusive questions:

*“I don't mean to pry – but is everything OK? You're looking quite… well, peaky!” (student 4)*

Goffman defined two levels of stigma – the discredited and the discreditable. A discreditable person is someone whose stigma has not yet been revealed, and may try to conceal it. Goffman terms this “passing” [26]. Passing often involves the management of information, with associated stress and distancing from others. Here, two different characters try to evade questioning:

*Mentally too tired to prepare answers, my tactic was to remain as unresponsive as possible, like a suspect without a lawyer. (student 4)*

*Let's try and bring the conversation round to something else… (student 6)*

A concept related to stigma is that of contagion, where people behave as though a non-infective disorder is catching. Here a character believes it literally:

“*Please, Dr sir, do not get too close. You are too kind. I do not wish to cause any harm to anyone else..” (student1)*

Falk introduced the concept of achieved stigma: “stigma that is earned because of conduct and/or because they contributed heavily to attaining the stigma in question” [34]. Here we see the neighbour of a character with hepatitis C putting her firmly into the achieved stigma category:

*“It’s your mother I feel most sorry for, you really have made it your life’s mission to destroy her.*” (student 4)

### Narratives dealing with Self- Image

Stigmatised people have to adjust their self-image in response to their “spoiled” identity [26]. Heatherton notes that many stigmatised people view themselves contemptuously, [35], as shown by this example of writing.

*I can’t bear the idea that my failings will be obvious to this person, that she will guess my diminished state. (student 2)*

Here, a student's character contemplates the change in appearance of his hands after chemotherapy:

*As my hand reaches for the doorknob, my phone rings.*

*“Hello”*

*It can't be the hospital again. Surely not.*

*“Mr Mulligan?”*

*I'm staring at my hand on the door.*

*“Yes, who is this?”*

*It doesn't seem to belong.*

*“This is Sergeant Daniels, from the police.”*

*It's not really my hand though, is it?*

*“I see. What can I do for you?”*

*It looks so foreign. I don't recognise it.*

*“There's been another incident at school. Your son is here with us at the station. We were hoping you could come down so we could sort this out.”*

*My hands were strong. Powerful. Not these disgusting, purple-shoed spiders. The discoloured nails, the bulging tendons, the sagging skin. Surely they could never belong to me? (student 5)*

### Narrative Wreckage

Frank points out that serious illness is a loss of the “destination and map” that had previously guided the ill person's life and introduces the concept of “narrative wreckage”, the “damage that illness has done to the ill person's sense of where she is in life and where she may be going” [21].

Here a student shows her character's response to her diagnosis of Hepatitis C:

*Just as I'd finally got my life back on track after years of being in the gutter, and now this....” (student 4)*

Frank categorises illness narratives into three categories: a) restitution narratives, which have the storyline:”Yesterday I was healthy, today I’m sick, but tomorrow I’ll be healthy again.” b) Chaos narratives in which there is no control and an absence of narrative order and c) quest narratives, which are defined by the ill person’s belief that something is to be gained from the experience [21]. Here is a chaos narrative, in which a student's character describes living with the uncertainty as to whether or not his cancer is in remission:

*“And how did it look?” Alexander is impatient. He doesn’t realise this is difficult for me. To speak about this.*

*“Inconclusive.”*

*“What does that mean?”*

*I sigh. “They don’t really know. I have to wait and see. Can’t do any more treatments at the moment, my body can’t handle it. Don’t think I’d want to, even if I could.” I see Alexander’s face darkens back to an emotionless mask. Now he really looks like me.*

*“Oh, so you’re going to die?” he asks bluntly.*

*“I have to wait and see. Maybe it’s cured, maybe it’s coming back. Should find out in a month or so.”*

*There is a pause. “Fuck, man, that kinda sucks.” Yes, I suppose it does kinda suck. “How do you feel about it?”*

*Feel? I don’t know how I feel about it. I don’t know if I do feel about it. (student 5)*

Here are more chaos narratives:

*Slowly, I drag my weak, heavy limbs and clouded brain out of bed, concentrating on each step. Down the damp corridor with peeling wallpaper towards the door. I can see the faint red blur of the postman's jacket distancing further away through the window pane. All that's left is a pizza delivery advert and a red slip lying on the floor that infuriatingly reads “sorry you were out”- as if to mock my feeble attempt at movement. I'm reminded again of the cd that got stuck; useless and frustrating. (student 4)*

*What illness I suffer from, I do not know. The pain of the illness cripples me, leaves me sleepless and wrecks havoc with my insides. I'm sure it is what killed my family. … how much longer until it gets me too? (student 1)*

*I try to disconnect, to lose myself in some daydream or fantasy, to let my mind take over like I used to before. But I can't. The grey sombre weather, the putrid reek of my fellow humans, the doctor's clinically sterile behaviour, the mind-numbing tiredness, the realisation that I have wasted my life have drained me. Hollowed me out. There is no longer enough left of me to put into my imagination. I'm stuck, shackled to a world where everything is lonely, pointless and mortal. (student 5)*

*My memory is a messy patchwork of recounted, retrospective observations, facts and suppositions formed by doctors and various witnesses. Even the insurance companies seem to own a greater understanding of the circumstances than I do myself. (student 2)*

As the module progressed, some of the students moved their characters on to other “scripts”

Here is a quest script, written after a character has discovered that his IBS is neither contagious nor fatal:

*“As the doctor turns to leave, an idea comes to my mind. A purpose to my life that I could fulfil.*

*“Dr, I want to help other people who suffer from my illness. I want to dedicate my life to making others aware that they have an illness and nothing else.” (student 1)*

Here is another quest narrative:

*“So, what are you going to do? Dammit, you might only have a few weeks left to live! Do you wanna go out with regrets?”*

*“What do you suggest I do?” I notice a twinkle in his eye. Were they always like that?*

*“When we get home, we’re writing a list.” He seems passionate.*

*“A list?”*

*“A list. Of all the things you regret not doing. And then we’re going to do them.” He is smiling now.*

*“We?”*

*“Yup. We.” He gets up and heads towards the cafes door.*

*“Don’t you have school?” I call out after him.*

*“Nah,” he responds without a backwards glance, “I’ve been suspended for trying to burn it down.”*

*I get up to follow him. He looks like me. But he is definitely Cassandra’s son. (student 5)*

Here is a restitution script:

*“Can I get you anything?” asks a young girl, tray of empty glasses balanced in one hand.*

*“Oh…a latte please”, I smile up at her.*

*“No problem.” she returns the smile and turns away. A normal exchange for her, a small victory for me. The thank you had slipped from my lips without a silent reminder, a reprogramming of expected pleasantries that was becoming natural once more. My lips had quirked into that smile of their own volition, no forced mimicry of expression that it once was. I'm winning.” (student 2)*

Frank suggests that finding a new story restores coherence and offers the potential for healing [21].

Here, a character contemplates a photograph of herself, taken before her head injury.

*“I am no longer her. …… it is hard to mourn your own death when you are still living….....The shy, smiling bride's hopes and dreams for the future no longer represent my own. I am changed, a different person. But I can see that I am alive. I must box up my old life, clear space for the endless potential that is me.*

*I smile at her captured image, celebrate her love, her happiness and bid her goodbye.” (student 2)*

### Comments relating to affinity

The students were deliberately not given a choice of the age, gender, social circumstances or disorder of the patient they were to write about. Some were not pleased to be allocated patients with socially stigmatising disorders, or who were homeless, and admitted that they found it difficult to relate to their character initially:

*“Initially I struggled to create an authentic character that I believed in – I had little knowledge of the impacts of hepatitis C from the patient’s perspective.” (student 4)*

*“My initial understanding of Rebecca (my character’s name) had a rather tragic flavour. My imagination struggled to comprehend the possibility of life with such a disability, was frustrated by the limitations it imposed and saddened by the notable losses that would result.” (student 2)*

Some of the students wrote in their reports that they had felt increased empathy towards their character as the course progressed:

*I realised when writing that Andrew’s depression was not a new occurrence. It was something he had struggled with for most of his life. (student 5)*

*Putting him through different scenarios and using different writing types helped me create more about his feelings, his character….Through this course I feel like I evolved with my character and get to know more and more about him through the writing exercises. (student 6)*

*At first it was very unusual writing about a character I hadn't completely made up myself; as I progressed, I felt that I understood my character more due to the chronicity of her condition. This allowed me to empathise more with the situations she would find herself in on a daily basis. (student 3)*

*I found his story distressing and the more I spent on my creative writing, the more empathy I felt for his experiences in life. (student 1)*

*As the course progressed, the character developed from an abstract concept to a fully-fledged concept. As he gained depth, he became more relatable, gaining a history, ambitions, motivations, regrets that I could also understand. Additionally, as more was revealed about him, there was more for him to lose if his cancer came out of remission. Understanding this ominous sense of panic while trying to maintain a normal life made it easier to understand some of his decisions which might otherwise not have made sense (student 5)*

*Creative writing enabled me to access an otherwise alien experience and perspective and as a result feel genuine empathy and compassion towards someone I may otherwise have been quick to judge …….I was interested to see how my character’s tone and attitude impacted on both the group’s and my own emotions towards her…. (student 4)*

*My original perception was that her plot would necessarily be a tragedy, yet by truly engaging with the character, I began to realise her strength and potential, was able to envisage her success… my final piece demonstrates the acceptance, respect, love and hope that has developed for Rebecca, and which, as a partner in the telling of her story, she began to feel for herself. (student 2)*

## Conclusion

The quality of writing produced by students on the SSC was high, and their writing was often very moving. Analysis of the writing showed that the students were able to produce successful portrayals of the day- to- day experience of living with a chronic disorder. Although they were given generic topics to write about, they also introduced a number of important illness-related themes including stigma, personal identity and narrative wreckage. Not every student wrote about all of these themes, but as all of the writing was discussed in the weekly workshops, all of them discussed the themes in the context of the fictional characters.

As well as demonstrating cognitive empathy, the ability to understand how another person feels, there was also some evidence of affective empathy, experiencing he emotions that other people are feeling (“*I found his story distressing”*, “*My final piece demonstrates the acceptance, respect, love and hope that has developed for Rebecca and which…she began to feel for herself”).* An intention to help is obviously harder to demonstrate in creative writing, but it could be argued that the students who moved their characters on from their chaos scripts were trying to help them.

A recurrent theme was that during the process of creating and then repeatedly writing about the same fictional character the students felt that they developed a deeper empathic understanding of their character, even when they initially felt little connection with them.

Gordon and Evans suggest that many students are “disadvantaged” by their material advantages when it comes to understanding other people’s lives [36]. It appears that in this SSC, writing repeatedly about a character they initially felt little affinity with encouraged students to feel more empathic towards their fictional characters. This is an important finding.

There has recently been interest in Longitudinal Integrated Clerkships (LICs). These are lengthy immersion placements, often in rural primary care, during which students have the opportunity to experience continuity of care and management of patients with chronic illness. They have been shown to produce more empathic students [37, 38]. However, many medical courses offer mainly short rotations during which there may be limited opportunities to get to know patients well. One of the key features of LICs is the opportunity get to know and follow up patients. In a similar manner, repeatedly writing about the same character enabled the students on the SSC to get to “know” their characters. Charon suggests that health care workers need to develop “narrative competence”, the ability to “recognise, absorb, interpret and be moved by the stories of illness” [29] and Kleinman has argued that “the interpretation of narratives of illness experience is “a core task in the work of doctoring, although the skill has atrophied in biomedical training” [28]. Getting to know the patient and their narrative is a necessary pre-requisite to this. The group experience of the SSC offered a safe and curated space without background “noise” within which the students could explore a patient narrative in depth, and offered the group the opportunity to learn from each other. Elizur and Rosenheim have previously demonstrated how group experience can consolidate and deepen genuine empathy, and found that the effect of this persisted at follow-up [39]. The “empathy effect” effect of LICs has also been shown to persist after qualification as a doctor [40]. Shapiro and colleagues found that students trained in point-of-view writing demonstrated significantly more awareness of emotional and spiritual aspects of a paper case in and end of year writing assignment than did students trained in clinical reasoning [41], while DasGupta and Charon found that a group of students who discussed their own personal illness narratives also reported an increased sense of regard and empathy for patients [42].

Kumagi has written about the transformative nature of learning involving the use of narratives. He suggests that it involves learning on cognitive, affective and experiential levels, and results in a shift in non-verbalised, habitual, taken-for-granted frames of reference towards a perspective that is more open, reflective, and capable of change [43]. Gottschall, pointing to the evidence of a link between fiction reading and good social skills, comments on the role of implicit memory, the memory that is inaccessible to the conscious mind. He points out that realistic rehearsal of any skill leads to enhanced performance and that it is an axiom of neuroscience that “cells that fire together wire together” [44], a process that may also be applicable to rehearsal of empathy. It may not matter whether a narrative is constructed through creative writing or discovered through repeated clinical contact.

This study is based on a single module attended by six students and uses a qualitative approach to analyse whether the students were able to demonstrate empathy. The relatively small number of students involved means that the reports should be seen as a preliminary analysis, however the findings suggest that further work in this area is merited. There are challenges in considering how this should be done. Empathy is more commonly measured by self- administered questionnaire. However, as Pederson and Hemmerdinger and Stoddart have commented, there are issues of reliability and validity with most questionnaires attempting to measure empathy [45, 46]. A recent validation study using patient views found a correlation value of r = 0.48 for one questionnaire, [47] but a further attempt to validate the same questionnaire using feedback from standardised patients raised questions about possible ethnicity and gender biases in assessments carried out by the standardised patients [48].

As Gordon and Evan point out, medical humanities are rarely part of the mainstream curriculum in medical schools, and are generally undertaken on an opt-in basis by small groups of students, making quantitative research difficult. However, as Kumagai notes, “qualitative approaches yield a more in-depth, richer understanding of the meaning that individuals confer on events or life experiences” [43], while Greenhalgh notes that narrative techniques “provide the opportunity to generate insights that cannot be gained using the traditional tools of the quantitative researcher” [49] There may be a case to further develop qualitative work in this area.

Analysis of the students’ writing showed that they demonstrated an ability to “stand in another’s shoes” and, interestingly, the students’ comments on their own writing showed that their ability to empathise with characters they initially felt little affinity for deepened as the SSC progressed. A medical realism approach to creative writing, combined with creating and then repeatedly writing about the same fictional character, offers the potential for a low-cost intervention in a safe and convenient environment, which can potentially help to increase the empathic ability of students. As such, it merits further assessment, which may be most appropriately carried out by qualitative methods.

### Ethics/consent statement

This article is based on a poster developed by PM and the other students. The work has not been published in whole or in part before. All authors have been actively involved in contributing to the content of the article.

## Abbreviations

LICS: Longitudinal integrated clerkships
SSC: Student selected component

## References

[1] Shapiro J (2011). Perspective: does medical education promote professional alexithymia? A call for attending to the emotions of patients and self in medical training. Acad Med..

[2] Greaves D (2004). The Healing tradition: reviving the soul of Western medicine.

[3] Pink DH (2006). A whole new mind: why right brainers will rule the future.

[4] Shapiro J (2002). How do physicians teach empathy in the primary care setting?. Acad Med..

[5] Zachariae R, Pedersen CG, Jensen AB, Ehrnrooth E, Rossen PB, von der Maase H (2003). Association of perceived physician communication style with patient satisfaction, distress, cancer-related self-efficacy and perceived control over the disease. Br J Cancer..

[6] Hojat M, Louis DZ, Markham FW, Wender R, Rabinowitz C, Gonnella JS (2011). Physicians' empathy and clinical outcomes for diabetic patients. Acad Med..

[7] Kim SS, Kaplowitz S, Johnston MV (2004). The effects of physician empathy on patient satisfaction and compliance. Eval Health Prof..

[8] Blatt B, LeLacheur SF, Galinsky AD, Simmens SJ, Greenberg L (2010). Does perspective-taking increase patient satisfaction in medical encounters?. AcadMed.

[9] Hoffman MI (2000). Empathy and moral development: implications for caring and justice.

[10] Hojat M, Gonnella JS, Nasca TJ, Mangione S, Vergare M, Magee M. Physician empathy: Definition, components, measurement, and relationship to gender and specialty. Am J Psychiatry. 2002;159:1563–69. 10.1176/appi.ajp.159.9.1563 12202278

[11] Larson EB, Yao X (2005). Clinical empathy as emotional labour in the patient-physician relationship. JAMA.

[12] Garden R (2007). The problem of empathy: medicine and the humanities. New literary history.

[13] Fan Y, Duncan N, de Greck M, Northoff D (2011). Is there a core neural network in empathy? An fMRI based quantitative meta-analysis. Neuroscience & Biobehavioral Reviews.

[14] Corradini A, Antonietti A (2013). Mirror neurons and their function in cognitively understood empathy. Consciousness and Cognition.

[15] Jacob P (2008). What do mirror neurons contribute to human social cognition?. Mind and Language.

[16] Batt-Rawden SA, Chisholm MS, Anton B, Flickinger TE (2013). Teaching empathy to medical students: an updated systematic review. Acad Med.

[17] Morris P (2003). Realism.

[18] Rothfield L (1992). Vital Signs: Medical realism in nineteenth century fiction.

[19] Woolf V. On being ill. Martino Publishing 2014 (originally published in the New criterion 4 (1) 1926).

[20] Jurecic A (2012). Illness as a narrative.

[21] Frank AW (1995). The Wounded Storyteller. Body, Illness and Ethics.

[22] Green MJ, Myers KR (2010). Graphic medicine: use of comics in medical education and patient care. BMJ.

[23] Williams I (2012). Graphic medicine: comics as medical narrative. Medical Humanities.

[24] ONS Department for work and pensions office for disability issues. Disability facts and figures 16.1.2014 . Available from: https://www.gov.uk/government/publications/disability-facts-and-figures/disability-facts-and-figures Accessed 3.3.2015.

[25] 25. Health and Social Care Information Centre. Quality and Outcomes Framework (QOF) - 2013–14 Publication date: October 28, 2014. Available from: http://www.hscic.gov.uk/catalogue/PUB15751 Accessed 3.3.2015.

[26] Goffman E (1963). Stigma: the management of spoiled identity.

[27] Sontag S (2002). Illness as a metaphor.

[28] Kleinman A (1988). The illness narratives. suffering, healing and the human condition.

[29] Charon R (2006). Narrative medicine: Honoring the stories of illness.

[30] Stewart I, Joines V (1987). TA today: A new introduction to transactional analysis.

[31] Erasmus programme. Accessed at: http://erasmusprogramme.com/ Accessed 3.3.2015.

[32] Bryant A, Charmaz K, Bryant A, Charmaz K (2007). Grounded theory research; methods and practices. The Sage handbook of grounded theory.

[33] Kristiansson MH, Troein M, Brorsson A (2014). We lived and breathed medicine- then life catches up: medical students’reflections. BMC Medical Education.

[34] Falk G (2001). Stigma- how we treat outsiders.

[35] Heatherton T, Hebl MR, Hull JG, Kleck RE (2000). The social psychology of stigma.

[36] Gordon JJ, Evans HM (2007). Learning medicine from the humanities.

[37] Thistlethwaite J, Bartle E, AiLing Chong A, Dick ML, King D Mahoney S. BEME review: A Review of Longitudinal Community and Hospital Placements in Medical Education 2013. Available at: http://www.bemecollaboration.org/Published+Reviews/Longitudinal/ Accessed 4.3.2015. 10.3109/0142159X.2013.806981 23848374

[38] Walters L, Greenhill J, Richards J, Ward H, Campbell N, Ash J (2012). Outcomes of longitudinal integrated clinical placements for students, clinicians and society. Med Educ.

[39] Elizur A, Rosenheim E. Empathy and attitude among medical students: the effects of group experience. Acad Med.1982;57:675–83. 10.1097/00001888-198209000-00003 7202049

[40] Gaufberg E, Hirsh D, Krupat E, Ogur B, Pelletier S, Reiff D (2014). Into the future: patient-centredness endures in longitudinal integrated clerkship graduates. Med Educ.

[41] Shapiro J, Rucker L, Boker J, Lie D (2006). Point-of-view writing: a method for increasing medical students’ empathy, identification and expression of emotion, and insight. Education for Health.

[42] DasGupta S, Charon R (2004). Personal illness narratives: using reflective writing to teach empathy. Acad Med..

[43] Kumagi AK (2008). A conceptual framework for the use of illness narratives in medical education. Acad Med.

[44] Gottschall J (2013). The storytelling animal-how stories make us human.

[45] Pederson R (2009). Empirical research on empathy in medicine- a critical review. Patient education and counselling..

[46] Hemmerdinger J, Stoddart SD, Lilford RJ (2007). A systematic review of tests of empathy in medicine. BMC Medical Education.

[47] Glaser KM, Markham FW, Adler HM, McManus PR, Hojat M (2007). Relationships between scores on the Jefferson scale of physician empathy, patient perceptions of physician empathy, and humanistic approaches to patient care: a validity study. Med Sci Monit.

[48] Berg K, Blatt B, Lopreiato J, Jung J, Schaeffer A, Heil D (2015). Standardized patient assessment of medical student empathy: ethnicity and gender effects in a multi-institutional study. Acad Med.

[49] Greenhalgh T (2006). What seems to be the trouble? Stories in illness and healthcare.

